# Changes in stimulus envelope reveal two classes of peripheral electrosensory neurons

**DOI:** 10.1186/1471-2202-15-S1-P191

**Published:** 2014-07-21

**Authors:** Michael G Metzen, Maurice J Chacron

**Affiliations:** 1 Department of Physiology, McGill University, Montreal, QC, Canada; 2 Department of Physics, McGill University, Montreal, QC, Canada

## 

Natural sensory stimuli are characterized by time varying moments such as mean (first-order) and variance (second-order). While psychophysical studies have shown that second order attributes (the envelope) are critical for perception, how they are encoded in the brain remains largely unknown. Here we focused on envelope coding by peripheral electrosensory neurons (P-type afferents) in the weakly electric fish, *Apteronotus leptorhynchus*, using narrowband noise stimuli that where modulated by sinusoids in the frequency range between 0.05 and 10Hz. Envelopes are an essential feature of natural electrosensory stimuli. When two fish come within close proximity of one another, each animal will experience an amplitude modulation of its own electric signal that oscillates at the difference between the individual EOD frequencies. The envelope varies in time as the distance between two fish changes, and recent studies have shown that it primarily contains low temporal frequencies (<1 Hz). While primary afferents always increase their firing rates in response to an increase in EOD amplitude, we show that they can either increase or decrease their firing rates in response to an increase in the envelope provided that this increase is greater than their threshold. However, the gain and phase of afferent responses are independent of envelope frequency. Therefore, this study gives important insights as to how neural heterogeneities can influence responses to envelopes and might provide an answer as to why primary afferent baseline firing rates vary over such a large range.

